# The Efficacy and Acceptability of Flash Glucose Monitoring in Pregnant Women with Gestational Diabetes Mellitus: A Systematic Review

**DOI:** 10.3390/jcm13237129

**Published:** 2024-11-25

**Authors:** Franciszek Ługowski, Julia Babińska, Zofia Awiżeń-Panufnik, Ewelina Litwińska-Korcz, Magdalena Litwińska, Artur Ludwin, Paweł Jan Stanirowski

**Affiliations:** 1 1st Department of Obstetrics and Gynecology, Medical University of Warsaw, 02-015 Warsaw, Poland; s084958@student.wum.edu.pl (J.B.); stanirowski@gmail.com (P.J.S.); 2 Department of Obstetrics, Gynecology and Oncology, Medical University of Warsaw, 03-242 Warsaw, Poland

**Keywords:** flash glucose monitoring, continuous glucose monitoring, gestational diabetes mellitus, self-monitoring of blood glucose

## Abstract

**Background:** Gestational diabetes mellitus (GDM) occurs in approximately 9% of pregnancies, and proper glycemic control is of utmost importance in the prevention of GDM-associated obstetric complications. Flash glucose monitoring (FGM), a subtype of continuous glucose monitoring (CGM), offers intermittent blood glucose scanning and is considered a propitious alternative to the standard method of self-monitoring of blood glucose (SMBG). **Aim:** The aim of this review was to systematically assess the efficacy and acceptability of FGM in in pregnancies complicated by GDM. **Methods:** A systematic literature search was performed in the PubMed, MEDLINE, Scopus, and Cochrane databases. The review was conducted following the PRISMA guidelines, and the study protocol has been registered in the PROSPERO database with the registration number CRD42024545874. **Results:** A total of 872 articles were initially identified, 141 publications underwent an in-depth full-text analysis, resulting in 133 studies being excluded from further assessment. Eventually, eight studies were included in the analysis. **Conclusions:** The analysis revealed that FGM is a safe and efficient method of glycemic control in GDM. The majority of the studies consider its accuracy comparable to SMBG. Furthermore, FGM is well accepted by patients with numerous advantages in user-friendliness over SMBG.

## 1. Introduction

Gestational diabetes mellitus (GDM) is a metabolic antepartum condition occurring in approximately 9% of pregnancies [1]. Diagnosis of GDM can be made at any time of gestation, however, it usually presents in the late second/early third trimester of pregnancy and typically resolves after labor [2]. Parameters used for GDM diagnosis differ depending on the region of the world, however, in the majority of countries diagnosis is based on the results of 75 g Oral Glucose Tolerance Test performed between 24–28 gestational weeks, and according to the criteria defined by the World Health Organization [3].

Pregnancy is a state of increased insulin demand, and women’s pancreas inability to cope is causative of GDM [4]. In addition, certain hormones, such as human placental lactogen, corticotropin-releasing hormone or prolactin produced by the placenta, are responsible for the occurrence of insulin resistance and thus significantly contribute to GDM development [5]. Over the years, numerous adaptive mechanisms involved in the process of retaining optimal glucose concentration despite insulin resistance have been described, such as maternal beta cell proliferation or an increase in the production and secretion of insulin [6]. Nevertheless, the inability to adapt occurs often and is favored by maternal obesity, low physical activity, or advanced age [7].

Maternal hyperglycemia leads to the increased secretion of fetal insulin and subsequently to excessive intrauterine fetal growth and macrosomia [8]. This may result in short- and long-term complications for newborns and children, such as birth trauma, neonatal hypoglycemia, and impaired glucose tolerance, as well as increased susceptibility to overweight and obesity in early childhood and adolescence [9,10]. Moreover, women with GDM-complicated pregnancies have a higher risk of Cesarean section, pregnancy-induced hypertension, and preeclampsia [11,12]. Of importance, it is well established that adverse perinatal outcomes correlate proportionally with the level of maternal dysglycemia [13,14]. Therefore, glycemic control is of utmost importance in the management of GDM [15].

Current options for glycemic control in pregnancy include self-monitoring of blood glucose (SMBG) and continuous glucose monitoring (CGM), which comprises real-time continuous glucose monitoring (rtCGM) and flash glucose monitoring (FGM). SMBG remains the cornerstone of GDM monitoring and is considered a safe and cost-effective method [16]. However, newer methods, such as CGM or FGM, have many unique advantages over SMBG. For instance, CGM offers constant glucose measurements, and both CGM and FGM do not require finger pricking, which is more patient-friendly and might increase compliance [17,18]. In addition, FGM offers immediate biofeedback with the possibility of quick observation and implementation of corrections [19]. The above-mentioned method involves the insertion of a factory-calibrated sensor into the subcutaneous fat tissue for up to 14 days, which intermittently scans the glucose levels in the interstitial fluid [20]. FGM has been approved for the monitoring of GDM since 2017, and numerous studies have proved its efficacy [21]. Being a more flexible method that eliminates the disadvantages of SMBG, such as pain or stigmatization, FGM might improve adherence to the clinical recommendations. Nonetheless, the use of FGM in the management of GDM is not well established and is mostly decided individually [22]. Currently, there are no systematically collected data on the use of FGM in the monitoring of GDM. As a result, the presented study aims to systematically assess the efficacy and acceptability of FGM, focusing on glycemic control and several perinatal outcomes.

## 2. Materials and Methods

### 2.1. Search Strategy and Study Selection

A systematic literature search was performed in the PubMed, MEDLINE, Scopus, and Cochrane databases in the period between 20 May and 25 June 2024. This review was conducted following the Preferred Reporting Items for Systematic Reviews and Meta-Analyses (PRISMA) guidelines, and the study protocol was registered in the International Prospective Register of Systematic Reviews (PROSPERO) registry (CRD42024545874). The search strategy consisted of combinations of free text and MeSH terms, including “flash glucose monitoring”, “flash glucose monitor”, “gestational diabetes mellitus”, “gdm”, “diabetes in pregnancy”, and “pregnancy”. Following the primary search, reference lists of selected studies were manually screened for other eligible publications. The inclusion criteria were randomized controlled trials and observational studies written in English. The exclusion criteria were studies regarding pregestational diabetes, other types of studies, such as animal studies, and studies written in languages other than English. Of importance, since “diabetes in pregnancy” was used as a search term, studies on pregestational diabetes were not considered relevant for the data synthesis. The risk of bias in the selected studies was assessed independently by three researchers (F.Ł., J.B., and Z.A-P.) using the Downs and Black Checklist [23]. Randomized controlled trials were only included if they achieved at least 24 out of 27 points, whereas for non-randomized studies, the score must have reached at least 11 out of 13 points. Following the initial screening, the preselected studies were further analyzed to assess final eligibility for the systematic review. No meta-analysis was performed due to significant disparity in the study population, duration, and monitoring devices used.

### 2.2. Data Extraction and Analysis

Titles and abstracts were screened independently by four researchers (F.Ł., J.B., E.L-K., and M.L.) and collected data were inserted into the PICO table (Table 1). The following information was collected: author’s first name, type of article, year of publication, type of FGM device, duration of FGM usage, number of patients included in the study, glycemic control, qualification for insulin therapy, incidence of nocturnal hypoglycemia, glycosylated hemoglobin concentration (HbA1c), gestational weight gain, fetal birth weight, patients’ attitudes, and acceptability of FGM.

### 2.3. Outcomes

The primary outcome was glycemic control. Numerous secondary outcomes were also analyzed, including qualification for insulin therapy, the incidence of nocturnal hypoglycemia, HbA1c concentration, gestational weight gain, and fetal birth weight, as well as patients’ attitudes and acceptability.

## 3. Results

**Figure 1:**
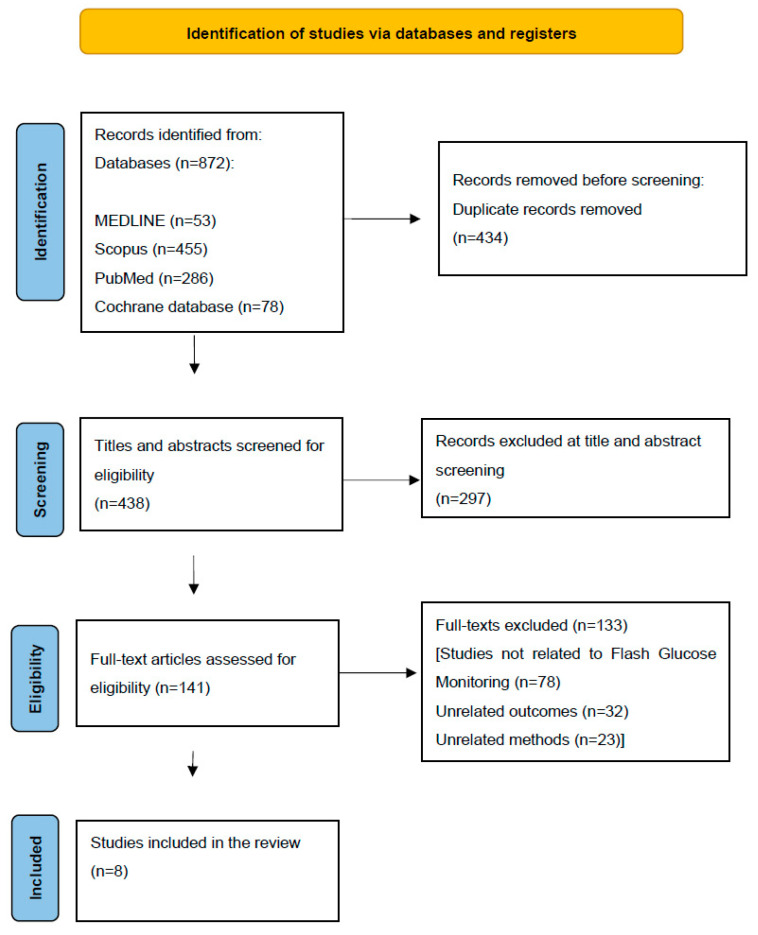
PRISMA flow diagram.

A total of 872 articles were identified through a systematic review of the literature (Figure 1). After initial screening, 434 duplicates were excluded, and 438 titles and abstracts were further screened to evaluate eligibility. A total of 141 publications underwent an in-depth full-text analysis, resulting in 133 studies being excluded from further assessment. Among those 133 excluded studies, 9 were disqualified based on bias assessment results. Eventually, a total of 8 publications were included in this systematic review (Table 2).

### 3.1. Glycemic Control

Six studies found the accuracy of FGM clinically acceptable for GDM management, including one reporting a high accuracy of 94.4% [25,26,27,28,29,30]. Pikee et al. found that FGM was able to pick the exact duration of hypoglycemia, which SMBG often missed [28]. Moreover, the minimum level of glucose detected by FGM was notably lower compared with SMBG (52.5 mg/dL vs. 72.7 mg/dL, *p* < 0.001). Citro et al. compared FGM with CG and revealed comparable results with a full concordance in the 70–110 md/dL glucose range [27]. In addition, the authors demonstrated that 68% of pairs of values measured by FGM were in the same glycemic range as capillary glucose. However, the agreement rate in the glucose range below 70 mg/dL was only 40%, with 60% of values indicated by the FGM being lower compared with capillary glucose. Lopes et al. revealed a high similarity of results between FGM and SMBG, reaching 94.4% [30]. Only one study found FGM’s accuracy insufficient and did not recommend it as the only method of glucose monitoring in GDM [31].

### 3.2. HbA1c

Two studies evaluated HbA1c concentration as an outcome. Majewska et al. reported no statistically significant difference in ΔHbA1c between the SMBG and FGM groups (0.05% and 0.1%, respectively, *p* = 0.546) [24]. The same study analyzed median HbA1C levels at the 1st, 3rd, and 4th visit in the period between 24 and 28 gestational weeks and delivery. No significant differences between both groups were noted (visit 1: FGM 4.9% vs. SMBG 4.9%, *p* = 0.782), (visit 3: FGM 5.1% vs. SMBG 5%, *p* = 0.409), (visit 4: FGM 5.1% vs. SMBG 5.1%, *p* = 0.802). Conversely, the second study showed significantly higher HbA1c levels at birth in the FGM group: 5.6% vs. 5.4%, *p* = 0.013 [25].

### 3.3. Insulin Therapy

Only one study assessed qualification for insulin therapy [24]. The publication revealed no significant difference with respect to the number of participants requiring pharmacologic treatment between the FGM and SMBG groups (30.61% and 32%, respectively, *p* = 0.827) [24].

### 3.4. Gestational Weight Gain

Gestational weight gain was assessed in two studies [24,25]. Both studies found no statistically significant differences between the FGM and SMBG groups [24,25]. Majewska et al. reported a median GWG of 2 kg for the FGM group and 3 kg for the SMBG group; during the period from the recruitment (24–28 weeks) to the 3rd follow-up visit (34–36 weeks) *p* = 0.682 [24]. Bastobbe et al. reported mean GWG during total pregnancy period of 10.6 kg for the FGM group and 12 kg for the SMBG group, *p* = 0.573 [25].

### 3.5. Neonatal Outcomes

Neonatal outcomes were analyzed in three publications [24,27,28]. A significantly lower incidence of fetal macrosomia (4.08% vs. 20%, *p* = 0.028) among women applying FGM was found by Majewska et al. [24]. In addition, the above-mentioned study demonstrated a decrease in the rates of LGA neonates (20.41% vs. 30%, *p* = 0.643) and neonatal hypoglycemia (8.16% vs. 20%, *p* = 0.148); however, observed differences were not significant. Citro et al. reported an incidence of neonatal hypoglycemia of 16% in a group of 19 participants in whom FGM was applied during CS [27]. In addition, in the study by Pikee et al., mean fetal birth weight was 2.90  ±  0.62 kg (range: 0.6–3.9 kg), hypoglycemia occurred in eight (11.43%), respiratory distress syndrome in three (4.29%), hyperbilirubinemia in eight (11.43%) neonates, and two neonatal deaths (2.86%) were noted [28].

### 3.6. Patient Satisfaction

Four publications reported higher patient satisfaction while using FGM in comparison with SMBG [25,26,28,30]. Bastobbe et al. reported increased convenience, flexibility, and a lack of pain [25]. Scott et al. reported that 100% of women considered FGM less painful, and 95.2% found it more discreet than SMBG [26]. FGM was also preferred in an Indian study, in which it was considered “excellent” by 97.1% of participants [28]. No severe or unanticipated events occurred while using FGM [25,26,28,30].

### 3.7. Adverse Events

Two studies analyzed adverse events during FGM usage [26,30]. Lopes et al. reported mild adverse reactions, such as pruritus or erythema [30], while Scott et al. reported no adverse events at all [26].

## 4. Discussion

The aim of this systematic review was to assess the efficacy of FGM in glycemic control in pregnancies complicated by GDM. In general, the results of our study indicate that FGM is comparable to SMBG in dysglycemia detection and assessment. The majority of studies showed high and clinically acceptable accuracy of FGM [25,26,28,29,30]. One study reported major advantages of FGM regarding the detection of dysglycemia [28]. In that study, FGM outperformed SMBG by picking up hyperglycemia in a greater percentage of women than the latter [28]. Of importance, a single study reported a full agreement between the FGM and capillary blood glucose measurements for glucose values between 70 and 110 mg/dL [27]. Therefore, in the authors’ opinion, FGM could be safely used for guiding intrapartum glucose and insulin infusions [27]. Regarding episodes of hypoglycemia, two studies proved FGM to be an efficient method of detecting nocturnal hypoglycemia in addition to establishing the precise duration of hypoglycemia [24,28]. These results are coherent with studies indicating the superiority of FGM in detecting and reducing time spent in hypoglycemia in patients with type 1 and 2 diabetes mellitus [32,33,34]. On the other hand, a study by Citro et al. reported a lower accuracy of FGM in detecting hypoglycemia compared with capillary glucose, and Heindrichs et al. described an overall unsatisfactory accuracy of the FGM device [27,31]. In particular, the latter study revealed that FGM underestimated blood glucose levels, making it discriminatory for GDM management. According to the authors’ opinion, the obtained results indicate the possibility of FGM use only as an addition to SMBG in GDM-complicated pregnancies [31]. The above-mentioned disparities with regard to glycemia assessment might be a result of different methodologies used in the studies, for instance, the number of participants recruited, the version of the FGM device used, or the duration of the FGM application.

HbA1c levels, commonly used in diabetes management, did not differ significantly between the SMBG and FGM methods in one randomized controlled trial that analyzed it [24]. Moreover, no differences were observed with regard to ΔHbA1c between serial measurements [24]. On the contrary, Bastobbe et al. observed a significant difference in HbA1c at delivery and comparable ΔHbA1c values [25]. Although numerous studies have indicated that adverse perinatal outcomes can be predicted by HbA1c concentration in women with concomitant GDM, the inability of the marker to detect short-term glucose fluctuations represents a major limitation [35]. As a consequence, in the opinion of many authors, HbA1c cannot be considered the most reliable parameter in the management of GDM. In addition, different HbA1c cut-offs ranging from 5% to 6.5% have been used in previous publications to predict adverse neonatal outcomes in GDM-complicated pregnancies, making comparisons even more problematic [36,37,38].

Three studies analyzed neonatal outcomes among women applying FGM [24,27,28], but only one compared them with the SMBG [24]. Majewska et al. found a lower incidence of fetal macrosomia and LGA neonates in the FGM group [24]. In addition, neonatal hypoglycemia occurred less often in comparison with the SMBG group. Nonetheless, with regard to the latter two outcomes, differences were not statistically significant [24]. The remaining two studies evaluated neonatal outcomes among FGM users during CS exclusively or following a period of 5–14 days of simultaneous FGM/SMBG application [27,28]. Both studies reported neonatal hypoglycemia rates comparable to other methods of blood glucose monitoring, such as CGM [39,40]. In addition, in the latter study, neonatal birth weight, as well as the incidence of neonatal death, was similar to results observed in studies analyzing the use of CGM [39,40]. Hence, there is an important need for further research in the area to elucidate if the lack of statistically significant differences in neonatal outcomes is a result of methodological errors. Presumably, FGM should have a positive impact on neonatal outcomes, as some of the above-mentioned studies indicated its superiority over SMBG in the detection of hyper- and hypoglycemia [24,28].

Four studies report the superiority of FGM in terms of patient-friendliness [25,26,28,30]. Bastobbe et al. reported subjectively perceived advantages, such as safety, lack of pain, and greater flexibility. Of importance, the transition from SMBG to FGM was associated with meaningful relief due to more painless glycemic control. In addition, women reported the convenience and simplicity of the data transfer to their cell phones [25]. Also, Lopes et al. revealed highly favorable attitudes, with over 95.9% of patients considering FGM entirely painless and superior to SMBG in terms of user-friendliness [30]. Consequently, these outcomes indicate the significant advantage of FGM over other methods in patients’ acceptability.

Numerous areas for improvement need to be implemented for the validity of this review. Firstly, most of the analyzed studies included small study groups of GDM women, and only two studies recruited more than 100 participants [24,25]. Secondly, only one out of eight studies was a randomized controlled trial. Thirdly, not all the studies were conducted over a sufficiently long period, and only in a few studies a combined analysis of maternal and neonatal outcomes was performed [24,25,27,28]. Lastly, some of the assessed outcomes were evaluated in merely one or two studies, which disallows conclusive observations.The strengths of this review include the selection of studies from the four major medical databases and its novelty as the first systematic review evaluating FGM use in women with GDM, as well as the transparent inclusion and exclusion criteria.

## 5. Conclusions

The findings of this systematic review indicate that FGM is a safe and efficient method of glycemic control in women affected by GDM. Studies included in the analysis support the thesis that FGM is as accurate as SMBG in the obstetric population. In addition, literature data point out many significant benefits of FGM, such as lack of pain, convenience, or the ability to transfer the data to one’s cell phone. The neonatal outcomes in GDM-complicated pregnancies managed with FGM are also satisfactory. Our review suggests better results in this matter compared with SMBG; however, more research needs to be conducted in this area.

In conclusion, there is a significant need for further research regarding the use of FGM in the management of pregnancies with concomitant GDM to support and elaborate the existing evidence.

## Figures and Tables

**Table 1:** PICO table to determine the eligibility of the research question.

Population	Intervention	Comparison	Outcomes
Pregnant women with gestational diabetes mellitus	The use of flash glucose monitoring for glycemic control	Other methods of blood glucose monitoring, such as continuous glucose monitoring or self-monitoring of blood glucose	Primary outcome: glycemic control. Numerous secondary outcomes, such as gestational weight gain, fetal birth weight, or user acceptability

**Table 2:** Characteristics of studies included in the review.

Authors	Type of Study	Study Population	FGM Device	Duration of FGM Usage	Outcomes	Results
Majewska et al. [24]	randomized controlled trial	50 FGM patients and 50 SMBG patients	FreeStyle^®^ Libre™ (Abbott Diabetes Care, Alameda, CA, USA)	28 days starting from the 24–28th week of gestation	GWG, fasting and postprandial glucose concentration, nocturnal hypoglycemic events, ΔHbA1c concentration, qualification for insulin therapy, incidence of CS, fetal macrosomia and LGA fetuses, neonatal hypoglycemia	No significant difference in the mean fasting glucose concentration between the FGM and the SMBG group was noted—86.71 mg/dL and 85.10 mg/dL, respectively (*p* = 0.437). Mean postprandial glycemia was lower in the SMBG group (109.52 mg/dL vs. 113.94 mg/dL, *p* = 0.011). Fetal macrosomia occurred more frequently in the SMBG group (20% vs. 4.08%; OR 5.62, 95% CI 1.16–27.22). No significant differences in the GWG, incidence of CS, LGA fetuses and neonatal hypoglycemia, ΔHbA1c, and qualification for insulin therapy between both groups were observed. FGM revealed a mean incidence of 15 nocturnal hypoglycemic events per month.
Bastobbe et al. [25]	prospective observational study	37 FGM patients and 74 SMBG patients	not specified	not specified	GWG, HbA1c concentration, hypertensive disorders of pregnancy, incidence of preterm deliveries, CS and LGA fetuses	HbA1c levels at birth were higher in the FGM group (5.6% vs. 5.4%, *p* = 0.01). No significant differences in the GWG, hypertensive disorders, rate of preterm deliveries and CS, incidence of LGA fetuses, or the need for NICU admission were demonstrated.
Scott et al. [26]	prospective observational study	74 FGM patients	FreeStyle^®^ Libre	14 days	accuracy of the system, user acceptability	Study revealed good agreement between the sensor and CG values. Clinical accuracy of sensor results versus SMBG was demonstrated, with 88.1% and 99.8% of results within the Zone A and Zones A and B of the Consensus Error Grid, respectively. A total of 97.3% of participants considered FGM “comfortable to wear” and 100% expressed that “getting glucose readings from the sensor is less painful than getting glucose readings from finger pricks”.
Citro et al. [27]	prospective observational study	19 FGM patients	Freestyle^®^ Libre™ 2	during CS	mean glucose concentration, neonatal hypoglycemia	Study revealed good accuracy of FGM in most ranges of glucose concentrations compared with CG measurement. Incidence of neonatal hypoglycemia—16%.
Pikee et al. [28]	prospective observational study	70 patients applying FGM and SMBG simultaneously	FreeStyle^®^ Libre™ Pro	5–14 days	mean glucose concentration, duration of hypoglycemia, patient satisfaction, neonatal hypoglycemia, hyperbilirubinemia, respiratory distress syndrome and death rate	FGM was found to have better clinical utility to detect glycemic variability episodes and duration of asymptomatic or nocturnal hypoglycemia. FGM had the advantage of greater patient satisfaction than SMBG by avoiding repeated pricking, inconvenience and anxiety. Mean birth-weight was 2.90 ± 0.62 kg (range: 0.6–3.9 kg). Neonatal hypoglycemia was observed in 8 (11.43%) neonates, respiratory distress syndrome in 3 (4.29%), hyperbilirubinemia in 8 (11.43%), and 2 neonatal deaths (2.86%) occurred.
Milln et al. [29]	prospective observational study	28 FGM patients	FreeStyle^®^ Libre™	48–96 h	accuracy of FGM in the measurement of blood glucose concentration	The overall correlation between the FGM glucose levels and venous glucose for combined fasting, 1 h and 2 h concentrations was 0.81 (0.69–0.89).
Lopes et al. [30]	prospective observational study	24 FGM patients in the acceptability analysis and 19 in the accuracy analysis	FreeStyle^®^ Libre™	28 days	accuracy of FGM in the measurement of blood glucose concentration, user acceptability	FGM showed good performance in GDM with regard to accuracy and usability. Device was highly accepted by the patients.
Heindrichs et al. [31]	prospective observational study	14 FGM patients	FreeStyle^®^ Libre™ and others-not specified	between 39 and 64 days (mean 52 ± 7.7 days)	accuracy of FGM in the measurement of blood glucose concentration	Accuracy of the first version of FGM device was insufficient as it lacked accuracy in the detection of slight glycemic increases.

GDM—gestational diabetes mellitus; CG—capillary glucose; CS—Cesarean section; FGM—flash glucose monitoring; GWG—gestational weight gain; HbA1c—glycosylated hemoglobin concentration; ΔHbA1c—difference between HbA1c measurements; LGA—large-for-gestational-age fetus; NICU—neonatal intensive care unit; SMBG—self-monitoring of blood glucose.

## Data Availability

The datasets used and/or analyzed during the current study are available from the corresponding author upon reasonable request.
